# Level of Adherence to Glaucoma Medication and Its Associated Factors Among Adult Jordanian Patients

**DOI:** 10.7759/cureus.63475

**Published:** 2024-06-29

**Authors:** Ahmad A Alhusban, Mohannad Albdour, Ala A Alhusban, Ghadeer Alhumimat, Walid Al-Qerem, Abdel Qader F Al-Bawab

**Affiliations:** 1 Department of Ophthalmology, King Hussein Medical Center, Amman, JOR; 2 Department of Pharmacy, Al-Zaytoonah University of Jordan, Amman, JOR

**Keywords:** questionnaire, associated factors, ophthalmic, medication, adherence, glaucoma

## Abstract

Background: Glaucoma is considered the second most common cause of blindness in patients above the age of 50. Lack of adherence to glaucoma medications frequently results in undesirable complications, specifically blindness and disability.

Purpose: The study’s objectives are to evaluate the level of adherence to glaucoma topical medications and factors associated with adherence to glaucoma medications.

Patients and methods: In total, 348 patients, of whom 48.6% were above the age of 65, were recruited. A cross-sectional study from August 2018 to March 2020 was conducted on glaucoma patients who were referred to the Department of Ophthalmology in Royal Medical Services in Amman, Jordan. A questionnaire was employed to collect patients’ demographic data, level of adherence, and factors associated with medication adherence. The inclusion criteria include the following: age above 20 years, diagnosis of glaucoma, currently under medical treatment, and willingness to participate in the study. Exclusion criteria include the following: patients who were hospitalized for glaucoma treatment, patients who had unstable medical conditions, and any patients for whom ophthalmologists had determined that they should be excluded for any other reasons.

Results: Almost half (47.1%) of the patients adhered to their personal glaucoma medications, and the most frequent cause of nonadherence was forgetfulness (39.9%), whereas the least common was stopping the drug after feeling better (7.0%).

Conclusion: Proper patient education and explanation of the seriousness of medication adherence and its association with treatment outcomes, along with assisting old and disabled patients when applying ophthalmic medications, may positively improve the adherence of patients to glaucoma and other related visual impairment medications.

## Introduction

Glaucoma, an optic nerve damage that is progressive, can be irreversible and may result in blindness [1]. Glaucoma is globally the third most common cause of blindness, accounting for 14% of the entire blind population [2], and the second most common cause of blindness for patients above the age of 50, among whom 3.6 million blindness cases were reported due to glaucoma between 2010 and 2019 [3]. Open-angle glaucoma is considered the most common type of glaucoma [4]. Glaucoma has many risk factors such as intraocular pressure (IOP), family history, and age, which is the most significant risk factor in the rapid progression of glaucoma [5]. Moreover, emergent epidemiological evidence indicates that several chronic diseases, including hyperlipidemia, cardiovascular disease, hypertension, and diabetes, are associated with glaucoma [6]. However, the relationship remains controversial. In addition, glaucoma patients are notably more likely to have these diseases, and glaucoma itself can be a risk factor for systemic comorbidities, which might be life-threatening [7]. Hence, detecting the association between glaucoma and systemic comorbidities may help in preventing the development of glaucoma.

Adherence is generally described as “the degree to which the person’s behavior corresponds with the agreed recommendations from a health care provider” [8]. That said, factors contributing to adherence to medication and its associated factors are categorized mainly into four groups, which are medication-related, patient-related, provider-related, and environment-related factors [9]. Patients’ adherence to medication is a major challenge for successful treatment, which may help in preventing the development of disease complications [10]. Poor adherence is a widespread phenomenon that represents a major challenge for healthcare providers because it compromises the treatment effectiveness, health economics, and quality of life [10].

Several researchers have evaluated the prevalence of patients’ adherence to glaucoma medication and reported wide variations in adherence levels ranging from 35.5% to 72.7% [11,12]. Improving patients’ adherence to glaucoma medications has the potential to diminish the number of surgical interventions needed for glaucoma treatment, avoid unnecessary vision loss, and save money for the entire healthcare system [12,13]. Although the prevalence of nonadherence among glaucoma patients is well documented in the literature [14,15], such studies in Jordan and the Middle East are still limited.

Consequently, in this study, we intended to assess the level of glaucoma medication adherence and factors associated therewith among adult glaucoma patients in Jordan. In addition, we examined the different variables affecting glaucoma in Jordan, which may help to establish a preventive and effective treatment.

## Materials and methods

Study design and patients

A total of 348 patients attending the Department of Ophthalmology at the Royal Medical Services in Amman, Jordan, between August 2018 and March 2020, who met the inclusion criteria, were enrolled in this cohort observational, single-center study. The study was approved by the Royal Medical Services Human Research Ethics Committee (approval No. 9/1/2018). The following inclusion criteria were applied: patients who were aged above 20 years, diagnosed with glaucoma, under medical treatment, and willing to participate in the study. However, patients who were hospitalized for glaucoma treatment, those with unstable medical conditions, and any patients who were deemed unsuitable by ophthalmologists for other reasons were excluded from the study. Informed consent was obtained from all individual participants included in the study. All patients were interviewed and asked to fill out the questionnaire in a private room in the ophthalmology department.

The questionnaire used in this study was previously validated [16], and the validated Arabic form of the four-item medication adherence scale (4-IMAS) was applied using “Yes” or “No” responses [17]. The adherence score was calculated by the sum of the responses for the four items, in which a “Yes” response was given a point of one and a “No” response was given a point of zero.

Statistical analysis

All statistical analysis was performed using SPSS version 27 (IBM Corp., Armonk, NY). Categorical variables were expressed as frequencies and percentages, whereas continuous variables were expressed as means and standard deviations. To assess the internal consistency of the adherence, Cronbach’s alphas were computed. A Cronbach’s alpha value above 0.5 was considered acceptable [18] because lower values are common in binary data and with low numbers of questions [19,20]. Chi-square and Mann-Whitney U tests were applied to evaluate the variables’ association with adherence level. For the multivariate analysis, a binary regression was applied to identify variables associated with adherence levels among the study participants. The model included adherence level as a dependent variable and predictors with p < 0.02 in the univariable analysis as independent variables. Significance was determined at p < 0.05.

The severity of glaucoma was graded according to visual field defects and was classified as mild (mean deviation ≥ -6 dB), moderate (mean deviation < -6 but > -12 dB), and severe (mean deviation ≤ -12 dB) according to Hodapp-Anderson criteria [21].

## Results

The current study enrolled 348 glaucoma patients, with 48.6% of them being above the age of 65 years. Half of the participants (51.4%) were men, and 79.6% of them were unemployed. About 33.6% of the patients had glaucoma surgery, and 17.2% had a family history of glaucoma disease. Moreover, 79.6% of the participants had completed high school or less, and 37.4% of the participants required assistance when applying their eye drops. Participant demographic characteristics solely are summarized in Table 1.

**Table 1:** Sample demographic characteristics

Variables			Frequency (%)
Sex	Male		179 (51.4%)
	Female		169 (48.6%)
Age	>65 years		169 (48.6%)
	<65 years		179 (51.4%)
Employment	Employed		71 (20.4%)
	Unemployed		277 (79.6%)
Underwent glaucoma surgery			117 (33.6%)
Family history of glaucoma disease			60 (17.2%)
Does someone help you when applying your eye drops? (Yes)			130 (37.4%)
Education level		High school or less	277 (79.6%)
		Bachelor’s	59 (17%)
		Postgraduate	12 (3.4%)

The majority of the participants had open-angle glaucoma (73.3%), and 83.3% had the condition for more than a year. The most common comorbidity reported by the participants was hypertension (47.4%) followed by diabetes mellitus (32.5%). Prostaglandins and alpha 2-agonists were the most frequently used medications (75.3% and 75%, respectively), while the least commonly used medication was cholinergic agonists (0.6%). The study participant’s medical profiles are summarized and presented in Table 2.

**Table 2:** Medical profile of the study participants

Variables		Frequency (%)
Disease severity level	Mild	74 (21.3%)
	Moderate	125 (35.9%)
	Severe	149 (42.8%)
Type	Open angle	255 (73.3%)
	Other	93 (26.7%)
Comorbidity	Hypertension	165 (47.4%)
	Diabetes mellitus	113 (32.5%)
	Cardiovascular disease (CVD)	28 (8%)
	Other	26 (7.5%)
	Any comorbidity	207 (59.5%)
Glaucoma duration	<1 year	58 (16.7%)
	>1 year	290 (83.3%)
Medical history	Alpha 2-agonist	261 (75%)
	Beta-blocker	251 (72.1%)
	Prostaglandin	262 (75.3%)
	Cholinergic agonist	2 (0.6%)
	Carbon anhydrase inhibitor	242 (69.5%)

High adherence was reported by less than half of the participants (47.1%) as shown in Table 3. The most frequent cause of nonadherence was forgetfulness (39.9%), whereas the least common was stopping the drug after feeling better (7.0%; Cronbach’s alpha = 0.542).

**Table 3:** The four-item medication adherence scale

Statements	Mean (±SD) or frequency (%)
Ever forgetting to take medicines (Yes)	148 (39.9%)
Ever being careless about taking medicines (Yes)	57 (15.4%)
Stop taking medicines when feeling better (Yes)	26 (7.0%)
Stop taking medicines if you feel worse (Yes)	93 (25.1%)
Total score	0.89 (±1.03)
Adherence level	
Low	184 (52.9%)
High	164 (47.1%)

Variables that were significantly associated with the level of adherence included taking carbonic anhydrase inhibitors (p-value = 0.024), presence of other comorbidities (p-value < 0.001), someone helping the patient when applying the eye drops (p-value = 0.001), and patient's age group (p-value < 0.001) as provided in Table 4.

**Table 4:** Association of variables with the adherence levels as evaluated by the Chi-square/Mann-Whitney U test * Significant at p-value less than 0.05. ** Significant at p-value less than 0.001.

Variables		Adherence level		Chi-square (p-value)/Mann-Whitney U (p-value) test
		High adherence (frequency (%)/mean ± SD)	Low adherence (frequency (%)/mean ± SD)	
Sex	Male	81 (49.4%)	98 (53.3%)	0.52 (0.47)
	Female	83 (50.6%)	86 (46.7%)	
Age (years)**	>65	60 (36.6%)	109 (59.2%)	17.82 (<0.001)
	<65	104 (63.4%)	75 (40.8%)	
Employment	Employed	31 (18.9%)	40 (21.7%)	0.43 (0.51)
	Unemployed	133 (81.1%)	144 (78.3%)	
Underwent glaucoma surgery	Yes	51 (31.1%)	66 (35.9%)	0.89 (0.35)
	No	113 (68.9%)	118 (64.1%)	
Family history of glaucoma disease	Yes	27 (16.5%)	33 (17.9%)	0.13 (0.71)
	No	137 (83.5%)	151 (82.1%)	
Does someone help you when applying your eye drops?*	Yes	76 (46.3%)	54 (29.3%)	10.70 (0.001)
	No	88 (53.7%)	130 (70.7%)	
Education level	High school	128 (78%)	149 (81%)	0.80 (0.67)
	Bachelor's	29 (17.7%)	30 (16.3%)	
	Postgraduate	7 (4.3%)	5 (2.7%)	
Disease severity level	Mild	34 (20.7%)	40 (21.7%)	0.06 (0.97)
	Moderate	59 (36%)	66 (35.9%)	
	Severe	71 (43.3%)	78 (42.4%)	
Comorbidities**	No	88 (53.7%)	53 (28.8%)	22.23 (<0.001)
	Yes	76 (46.3%)	131 (71.2%)	
Beta-blocker	No	41 (25%)	56 (30.4%)	1.27 (0.26)
	Yes	123 (75%)	128 (69.6%)	
Glaucoma duration	<1 year	30 (18.3%)	28 (15.2%)	0.59 (0.44)
	>1 year	134 (81.7%)	156 (84.8%)	
Prostaglandin	No	35 (21.3%)	51 (27.7%)	1.90 (0.17)
	Yes	129 (78.7%)	133 (72.3%)	
Alpha 2-agonist	No	41 (25%)	46 (25%)	<0.001 (1.00)
	Yes	123 (75%)	138 (75%)	
Carbon anhydrase inhibitor*	No	41 (25%)	65 (35.3%)	4.37 (0.04)
	Yes	123 (75%)	119 (64.7%)	
Glaucoma type*	Open-angle	130 (79.3%)	125 (67.9%)	5.69 (0.02)
	Other	34 (20.7%)	59 (32.1%)	
Number of eye drops		2.82 (± 1.18)	3.04 (± 1.15)	1696 (0.07)

Binary regression was conducted to evaluate the unique association between the independent variables and adherence level (Table 5). The results indicated that having someone to help the patient administer the eye drops (p-value = 0.002, OR = 2.146, (95% confidence interval (CI) = 1.324-3.480)) and having open-angle glaucoma (p-value = 0.001, OR = 2.455, (95% CI = 1.412-4.269)) significantly increased the odds to be in the high adherence group. On the other hand, having comorbidity (p-value < 0.001, OR = 0.419, (95% CI = 0.259-0.677)) and patients aged above 65 years (p-value < 0.001, OR = 0.369, (95% CI = 0.226-0.604)) were associated with a decrease in the likelihood to be in the high adherence group.

**Table 5:** Binary regression of the association of variables with the adherence levels

Variables		p-value	OR	95% OR	
				Lower	Upper
Number of eye drops		0.952	0.983	0.558	1.732
Comorbidities	Yes vs. No	<0.001	0.419	0.259	0.677
Does someone help you when applying your eyedrops?	Yes vs. No	0.002	2.146	1.324	3.480
Age (years)	>65 vs. <65	<0.001	0.369	0.226	0.604
Carbon anhydrase inhibitor	Yes vs. No	0.135	1.935	0.814	4.599
Prostaglandin	Yes vs. No	0.459	1.369	0.595	3.150
Beta-blocker	Yes vs. No	0.840	1.095	0.454	2.639
Underwent glaucoma surgery	Yes vs. No	0.412	0.810	0.489	1.340
Glaucoma type	Open-angle vs. Other	0.001	2.455	1.412	4.269

## Discussion

Glaucoma is one the most common diseases globally that may lead to severe complications including blindness [22]. Lack of adherence to medications for glaucoma remains a significant obstacle that hinders the control of glaucoma medication in millions of patients [23]. To the best of our knowledge and research, this is one of the few studies assessing the adherence levels among Jordanian glaucoma patients and their associated factors.

In the present study, we applied the four-item scale to evaluate adherence levels in glaucoma patients. This study showed that the adherence level of the participants was 47.1%. This is higher than the reported adherence levels in a study conducted in Iran (32.5%) [11]. However, the adherence level reported in the present study remains less than those reported in developed countries such as Canada (72.1%) [24] and the Netherlands (72.7%) [12]. These variations in adherence levels may be due to sociodemographic differences between the different countries in addition to variations in studies’ methodologies. Hence, interventions to increase adherence to medications might be required [25]. The most frequent reason for nonadherence in our study was medication forgetfulness (39.9%), which is consistent with other studies conducted in many countries [26,27]. The second reason was stopping medication when feeling worse, which was reported by 25.1% of the patients.

The most common type of glaucoma in our study was open-angle glaucoma (OAG), which was reported by 73.3% of the patients. This finding is consistent with studies conducted in Western countries [28]. Consistent with previous studies [29], almost half of the participants in the current study had severe glaucoma. Glaucoma in most cases is asymptomatic and often detected at a late stage [29]; researchers have found that half of the newly diagnosed glaucoma patients in developed countries were not aware of their disease [29]. As a result, large numbers of participants had severe glaucoma at initial diagnosis. Moreover, in total, 33.6% of the patients in the current study had undergone surgery as a treatment for glaucoma. This is an indicator of the progression of the disease, in which topical medication was not successful in controlling the medical condition.

Medications that lower IOP in the treatment of glaucoma are classified into alpha agonists, beta-blockers, prostaglandins, cholinergic agonists, and carbonic anhydrase inhibitors. The most frequently used medications in our study were alpha agonists and prostaglandins, whereas the least common were cholinergic agonists. This may be due to the highly reported side effects of the latter, such as blurred vision [30].

We reported that hypertension was the most common comorbidity among the patients. The significance of the association has been supported by some studies [31,32], whereas other studies did not demonstrate any significant association between hypertension and glaucoma [33]. This may be due to sampling variation and research methodology. The second most common comorbidity in our study is diabetes mellitus. This is consistent with a study in Jordan on the prevalence of glaucoma among diabetic patients, which stated that 50% of the diabetic patients who participated in the study had glaucoma [34]. This may be due to the increased glucose levels, which induce a high osmotic gradient that attracts fluids into the intraocular space, resulting in increased intraocular pressure. Moreover, the current study evaluated the association between several variables and adherence levels for glaucoma medications and reported significant associations with age, other comorbidities, and assistance in applying the ophthalmic medications.

Contradictory findings are reported in relation to other comorbidities and adherence levels in the literature. In one study, it was reported that other comorbidities were significantly associated with higher adherence [35], whereas in another, no relation was found between other comorbidities and adherence level [36]. This study revealed a significant association between the decreased level of adherence and other comorbid conditions. Similar findings have been reported in several studies that evaluated adherence levels in patients with other chronic diseases such as diabetes [37] and hypertension [38], which might be associated with more complex disabilities, frequency of medication, and therapeutic regimen complexity [17,39].

In agreement with a study conducted in Pakistan [40], when analyzed by age groups, adherence levels decreased in older patients, which suggests that impairment of physical and cognitive function in the elderly resulted in poor medication adherence such as missing drug doses. Furthermore, there are differences in adherence to ophthalmic drugs and oral drugs because the correct administration of ophthalmic drugs is more challenging when compared with oral medications, which requires ideal coordination between the psychomotor components of the human body [40]. This may also explain the association reported in the present study between adherence level and receiving assistance when applying ophthalmic medications. This was confirmed in an Iranian study, which reported that lack of family support was associated with a decreased level of adherence among glaucoma patients [11]. Therefore, it is highly important, particularly for elderly patients, to be supported by a member of their family to encourage them to take their medications on time and to help them with ophthalmic medication application.

Limitations of the study

The current study is one of the few studies to evaluate adherence levels among patients on glaucoma medications and its associated factors in Jordan and the Middle East. A limitation of the current study was that adherence level was self-reported, which may be influenced by recall and social desirability biases; nevertheless, the reported adherence prevalence is comparable to those reported in other studies. Another limitation of the current study is that we did not evaluate other variables that may influence patients’ adherence to glaucoma medications, including patients’ disease knowledge.

## Conclusions

This study revealed the average adherence rate to ophthalmic medication in patients with glaucoma in Jordan and identified modifiable factors related to poor adherence, including the need for assistance. Medical health professionals can play an important role in explaining the patients the importance of medication adherence and its association with treatment outcomes. Assisting aged and disabled patients when applying ophthalmic medication can significantly improve patients’ adherence levels. The primary reason for the lack of adherence was forgetfulness, and the second reason was stopping medication when feeling worse. Therefore, providing a simpler medication regimen with combined medications may significantly improve patients’ adherence.
